# Analysis of functional variants in mitochondrial DNA of Finnish athletes

**DOI:** 10.1186/s12864-019-6171-6

**Published:** 2019-10-29

**Authors:** Jukka Kiiskilä, Jukka S. Moilanen, Laura Kytövuori, Anna-Kaisa Niemi, Kari Majamaa

**Affiliations:** 1 Research Unit of Clinical Neuroscience, Neurology, University of Oulu, P.O. Box 5000, FI-90014 Oulu, Finland; 2 Department of Neurology and Medical Research Center, Oulu University Hospital, Oulu, Finland; 3 PEDEGO Research Unit, Medical Research Center Oulu, University of Oulu, Oulu, Finland; 4 Department of Clinical Genetics, Oulu University Hospital, Oulu, Finland; 5 Division of Neonatology, Rady Children’s Hospital San Diego, University of California San Diego, San Diego, California USA

**Keywords:** mtDNA, Athletic performance, Oxidative phosphorylation, Glycolysis, Selection

## Abstract

**Background:**

We have previously reported on paucity of mitochondrial DNA (mtDNA) haplogroups J and K among Finnish endurance athletes. Here we aimed to further explore differences in mtDNA variants between elite endurance and sprint athletes. For this purpose, we determined the rate of functional variants and the mutational load in mtDNA of Finnish athletes (*n* = 141) and controls (*n* = 77) and determined the sequence variation in haplogroups.

**Results:**

The distribution of rare and common functional variants differed between endurance athletes, sprint athletes and the controls (*p* = 0.04) so that rare variants occurred at a higher frequency among endurance athletes. Furthermore, the ratio between rare and common functional variants in haplogroups J and K was 0.42 of that in the remaining haplogroups (*p* = 0.0005). The subjects with haplogroup J and K also showed a higher mean level of nonsynonymous mutational load attributed to common variants than subjects with the other haplogroups. Interestingly, two of the rare variants detected in the sprint athletes were the disease-causing mutations m.3243A > G in *MT-TL1* and m.1555A > G in *MT-RNR1*.

**Conclusions:**

We propose that endurance athletes harbor an excess of rare mtDNA variants that may be beneficial for oxidative phosphorylation, while sprint athletes may tolerate deleterious mtDNA variants that have detrimental effect on oxidative phosphorylation system. Some of the nonsynonymous mutations defining haplogroup J and K may produce an uncoupling effect on oxidative phosphorylation thus favoring sprint rather than endurance performance.

## Background

Prolonged muscle activity in aerobic endurance performance requires sustained supply of energy that is provided in the form of adenosine triphosphate (ATP) [1]. Most of ATP is produced by oxidative phosphorylation (OXPHOS), where the transfer of electrons through four enzyme complexes (I-IV) and two electron carriers leads to a formation of proton gradient across the inner mitochondrial membrane. The gradient is then employed by complex V, ATP synthase, to generate ATP [2]. Short and high intensity efforts, such as that in sprint/power sports or in team sports, rely more on anaerobic glycolysis rather than OXPHOS.

The subunits of OXPHOS complexes are encoded in part by mitochondrial DNA (mtDNA) that harbors genes for 13 subunits as well as 22 tRNAs and two rRNAs [3]. Maternal inheritance, high mutation rate and lack of recombination have led mutations to accumulate sequentially in mtDNA lineages during population history. The ensuing groups of related haplotypes are continent-specific, e.g. Europeans harbor haplogroups H, V, U, K, T, J, W, I and X [4]. We have previously found that the frequencies of mtDNA haplogroups J and K are higher in Finnish sprinters than in Finnish endurance athletes and that none of the endurance athletes harbored haplogroup K or subhaplogroup J2 [5]. Such results prompted us to suggest that these mtDNA lineages could be “uncoupling genomes”. In mitochondrial uncoupling, electron transportation is uncoupled from energy production so that heat is generated instead of ATP [6]. Hence, “uncoupling genome” would be detrimental for endurance athletic performance. Consistent with our findings, Polish male endurance athletes harbor haplogroup K less frequently than the controls [7], and Iranian athletes representing power events or team sports have a higher frequency of haplogroup J than the controls [8]. Indeed, it has been shown that men with haplogroup J have lower maximal oxygen consumption than men with non-J haplogroups [9]. Together these findings suggest that haplogroup J rather than just subhaplogroup J2 and haplogroup K are candidates for being “uncoupling genomes”.

Most of the variants in mtDNA do not affect mitochondrial function. Unlike such neutral variants, non-neutral variants may have functional consequences and their effect on mitochondrial metabolism may be deleterious, mildly deleterious or beneficial [10]. Deleterious mutations cause OXPHOS defect and decline in ATP production and lead to variable disease phenotypes [11]. Combinations of mildly deleterious mtDNA mutations may confer a risk for complex diseases and phenotypes [12, 13]. In addition, beneficial nonneutral variants may become enriched in the population by adaptive selection [4]. Beneficial variants could affect elite athletic performance by increasing OXPHOS coupling efficiency and possibly provide an explanation, why certain mitochondrial lineages may be more favorable for endurance athletes than others.

Here we have analyzed entire mtDNA sequences from 141 Finnish elite athletes in order to study, whether the frequency of functional variants or whether the mutational load differ between the athletes and controls. In addition, the complete sequences enabled us to search for possible uncoupling variants within haplogroups J and K.

## Results

We determined complete mtDNA sequences of 141 Finnish athletes. These sequences and 77 sequences from control subjects were then used to generate a comprehensive phylogeny of 218 Finnish mtDNAs (Additional file 1: Figure S1). The athletes harbored 604 functional variants (rare variants, 28%) and the controls harbored 323 functional variants (rare variants, 23%). Altogether, there were 103 different rare variants including 65 nonsynonymous, 12 tRNA and 26 rRNA variants (Additional files 2, 3 and 4: Tables S1, S2 and S3). Quite strikingly, among the sprint athletes one of the rare variants was the pathogenic m.3243A > G mutation in *MT-TL1* and one was the pathogenic m.1555A > G mutation in *MT-RNR1*. The m.3243A > G mutation was heteroplasmic at a rate of 43% and the m.1555A > G mutation was homoplasmic.

The distribution of rare functional variants and common functional variants differed between endurance and sprint athletes and the controls (*p* = 0.04, X^2^ test). The difference appeared to be due to a higher number of rare functional variants among endurance athletes (Table 1). Mutational load of nonsynonymous variants and rare nonsynonymous variants did not differ between the groups (Additional file 5: Table S4).

**Table 1:** Mean number of functional variants per subject in Finnish athletes and controls

Type of substitution	Endurance (*n* = 52)	Sprint (*n* = 89)	Control (*n* = 77)
Number of common functional variants			
Nonsynonymous	0.94 ± 1.35	1.27 ± 1.57	1.30 ± 1.51
tRNA	0.54 ± 0.73	0.46 ± 0.60	0.60 ± 0.63
rRNA	1.46 ± 0.67	1.44 ± 0.72	1.35 ± 0.66
Total	2.94	3.17	3.25
Number of rare functional variants			
Nonsynonymous	0.83 ± 0.96	0.63 ± 0.91	0.61 ± 0.80
tRNA	0.19 ± 0.45	0.10 ± 0.30	0.14 ± 0.35
rRNA	0.37 ± 0.74	0.36 ± 0.73	0.19 ± 0.54
Total	1.39	1.09	0.94

Values are means ± standard deviations

We have previously shown that haplogroups J and K are infrequent among Finnish endurance athletes compared to sprinters or control population [5]. Here we determined, whether these haplogroups differ in sequence variation from that in the remaining mtDNA haplogroups among 218 Finnish subjects consisting of athletes and controls. Analysis revealed that the ratio between rare functional variants and common functional variants in haplogroups J and K was 0.42 of that in the remaining haplogroups (*p* = 0.0005, X^2^ test). In line with this, common nonsynonymous variants were more frequent in haplogroups J and K than those in the remaining haplogroups (Table 2). The subjects with haplogroup J and K also showed a higher mean level of nonsynonymous mutational load attributed to common variants than subjects with the other haplogroups, while the mutational load attributed to rare nonsynonymous variation was similar between haplogroups J and K and the remaining haplogroups (Additional file 6: Table S5).

**Table 2:** Mean number of functional variants per subject in haplogroups J and K and other haplogroups

Type of substitution	Haplogroups J and K (*n* = 22)	Other haplogroups (*n* = 196)	*P* value^1^
Number of common functional variants			
Nonsynonymous	3.64 ± 0.73	0.93 ± 1.30	< 0.000
tRNA	0.68 ± 0.57	0.51 ± 0.65	0.12
rRNA	1.41 ± 0.80	1.41 ± 0.68	0.98
Total	5.73	2.85	
Number of rare functional variants			
Nonsynonymous	0.68 ± 0.89	0.67 ± 0.89	0.92
tRNA	0	0.15 ± 0.38	0.053
rRNA	0.27 ± 0.46	0.31 ± 0.69	0.71
Total	0.95	1.13	

Values are means ± standard deviations. ^1^Mann–Whitney test. Samples from all three groups were pooled in order to maximize the number of sequences. Use of Finnish sequences was preferred over the use of sequences from the GenBank in order to avoid introduction of variation caused by population differences

## Discussion

We found differences in the distribution of rare functional variants in mtDNA between athletes and controls suggesting that endurance athletes harbor rare mutations that are beneficial for prolonged aerobic performance. We propose that such mutations could be favorable to the function of OXPHOS. Indeed, previously Japanese endurance athletes have been found to harbor a subset of mitochondrial DNA rare variants, clustered in branches of haplogroup A3, possibly influencing elite athletic performance [14]. It should be also noted that rare mtDNA variants have been associated with physiological and clinical phenotypes related with endurance performance including regulation of blood pressure [15], vascular function [16], body mass index and waist-hip ratio [17].

Non-neutral mutations in mtDNA may affect the function of OXPHOS and influence adaptation in varied energy demands. Adaptive mtDNA variants are less frequent in the population than the deleterious ones [18, 19], but animal studies have estimated that 26% of nonsynonymous substitutions are fixed by adaptive evolution [20]. Natural selection could favor retention of adaptive mutations enhancing OXPHOS and such mutations could be concentrated among endurance athletes, whose performance relies on efficient ATP production. Indeed, heterogeneous selection on OXPHOS genes have been detected among different fish species with extremes of high and low aerobic swimming performance [21]. Adaptive mutations could affect endurance performance by altering the expression of nuclear DNA. In keeping with this, mtDNA variants have been shown to be important modulators of autosomal disease [22].

Some of the rare nonsynonymous variants that were only harbored by endurance athletes (m.3308 T > C, m.5319A > T, m.9822C > T and m.12940G > A) show quite high probability of pathogenicity (> 0.4). The score suggests that these variants are at least function-altering. We do not consider that any of these rare variants alone, but rather rare variants as a group, could potentially affect OXPHOS. The status of m.3308 T > C as a disease-causing variant has been a matter of debate and haplogroup background could influence its penetrance [23]. Germline variants m.5319A > T, m.9822C > T and m.12940G > A, on the other hand, have not been reported as disease-causing in MITOMAP. Certainly, more studies will be needed to elucidate, if these variants have a beneficial effect on endurance capacity.

Previously, mtDNA mutations with high pathogenic potential have been detected in healthy human individuals in the 1000 Genomes Project and in individuals from the United Kingdom [24, 25]. However, to our knowledge, pathogenic mtDNA mutations have rarely been reported in elite athletes. Thus, surprisingly, two of the sprinters in our study harbored a disease-causing mtDNA mutation. One had the m.1555A > G mutation, a cause of hereditary nonsyndromic hearing loss [26], and the other had m.3243A > G, the common cause of the mitochondrial encephalopathy, lactic acidosis, stroke-like episodes syndrome (MELAS) [27]. The heteroplasmy of m.3243A > G was 43%, which is highly interesting, as age-adjusted m.3243A > G heteroplasmy in blood is as strongly associated with clinical disease burden and progression as muscle heteroplasmy levels [28]. Furthermore, heteroplasmy of > 40% in blood may lead to fully expressed MELAS phenotype [29]. The frequency of m.1555A > G in the population is 0.33% and that of m.3243A > G is 0.14% [30, 31], whereas the population frequencies estimated from patient cohorts are one tenth or less [32, 33]. This discrepancy suggests that there are unaffected or mildly affected subjects in the population. The finding that there were two sprinters with mutation suggests that sprint athletes may tolerate deleterious mtDNA mutations, while endurance athletes may not. Rather strikingly, given the above population frequencies and using the general formula for probability mass function, the probability of one and only one carrier of m.3243A > G among 89 subjects would be 11% and that of m.1555A > G would be 22%. These probabilities imply that the two mutations may have a beneficial, or at least not detrimental, effect for sprint performance. Indeed, sprint performance is based on anaerobic glycolysis rather than OXPHOS and [34], hence, mutations affecting OXPHOS would be less detrimental for sprinters than endurance athletes.

The rate of common nonsynonymous variation was higher in haplogroups J and K than that in the remaining haplogroups, but rare nonsynonymous variation was similar suggesting that the difference is due to nonsynonymous haplogroup-associated variants with minor allele frequency > 1%. The fact that only one endurance athlete belonged to haplogroup J and none to haplogroup K suggests that some of the nonsynonymous variants specific to these lineages may have a detrimental effect on endurance performance. Moreover, the high frequency of haplogroup J among centenarians and nonagerians has suggested that this haplogroup is beneficial for longevity [13, 35]. The reactions in OXPHOS produce a proton motive force across the inner mitochondrial membrane that is then harnessed in ATP formation. We have previously suggested the term “uncoupling genome” that would code for OXPHOS complexes that are less efficient in ATP production contributing to poor endurance performance and that produce lower amounts of reactive oxygen species contributing to longevity [5]. In the presence of an “uncoupling genome” the reactions dissipate the membrane potential favoring heat generation instead of ATP production. Indeed, experiments on human cell cybrids have shown that haplogroup J cybrids have lower levels of ATP and reactive oxygen species production than haplogroup H cybrids [36].

Electrons enter the mitochondrial respiratory chain primarily via complex I. Hence, the complex plays an essential role in generating mitochondrial membrane potential, determines the NADH/NAD+ ratio and is a major source of the reactive oxygen species [37]. Interestingly, two variants defining haplogroup J (m.4216 T > C, m.13708G > A) and m.3394 T > C occurring in haplogroup J are located in genes encoding subunits of complex I. These three mtDNA variants occur in the branches of European and Asian phylogeny indicating that they have arisen independently during evolution, i.e. are homoplasic, and suggesting that selective factors have favored their retention in the populations [38]. In addition, the variants are enriched in Tibetan highlanders and the Sherpas [39, 40], who are adapted to hypoxic environment.

Adaptation to ambient hypoxia brings about repression of mitochondrial respiration and induction of glycolysis. Recently, rather striking results have been seen in experimental mouse with inactivated *Ndufs4* gene that encodes another complex I subunit and leads to OXPHOS reduction. The ambient oxygen of 11% corresponding to 4000 m altitude resulted in amelioration of symptoms and longer survival compared to knock-out mice in atmospheric oxygen [41]. Our results, those showing that the frequency of rare variants in *MT-ND1* is higher in Japanese sprinters than that in controls [14], and population genetic and experimental data on adaptation and survival in hypoxic environment suggest that haplogroup J mtDNA or m.4216 T > C may reduce the capacity of OXPHOS and induce glycolytic pathway that would be beneficial for sprint performance. Furthermore, it is worth mentioning that some of the variants defining haplogroup J are located in the mtDNA regulatory region and may have functional importance. For instance, m.295C > T variant have been shown to impact mtDNA transcription and replication via in vitro transcription and cell culture studies [42]. Such variants could potentially enable quick transcriptional response to changing environmental conditions and stress, and thereby partially account for the functional impact of haplogroup J.

## Conclusions

Our results suggest that endurance athletes harbor an excess of rare mtDNA variants that may be beneficial for OXPHOS, while sprinters may tolerate mtDNA mutations that have disease-causing properties and have detrimental effect on cellular OXPHOS. Our previous finding on paucity of haplogroups J and K among endurance athletes was further examined by using complete mtDNA sequences. Common nonsynonymous variants were more frequent in haplogroups J and K compared to those in other haplogroups, suggesting that the uncoupling variants in haplogroups J and K are those defining these haplogroups. Indeed, the mutation load of these variants was considerably high, which increases the likelihood that some of these variants could alter function and negatively affect endurance performance. Our results are in line with previous studies indicating that at least some of the haplogroup-specific polymorphisms in mtDNA can have adaptive significance and common mutations in OXPHOS complex I genes are potential candidates to drive the functional impact of haplogroup J [4, 43–45].

## Methods

### Subjects and controls

Total DNA has previously been extracted from a national cohort of 141 Finnish track and field athletes including 52 endurance athletes (mean age, 21 ± 7 years; men, 26) and 89 sprinters (mean age, 20 ± 3 years; men, 45) [5]. Control mtDNA sequences (*n* = 77) were randomly selected from 192 Finnish sequences so that the proportions of mtDNA haplogroups matched with those in the population [46, 47]. The mean age of the sample population for the controls was 41 ± 12 years (men, 60%). Controls were not age-matched, as germline mtDNA variation remains unaltered throughout life.

### Molecular methods

The entire mtDNA coding sequence was determined by using a strategy consisting of conformation sensitive gel electrophoresis (CSGE) and subsequent sequencing (Big-Dye Terminator v1.1 Cycle Sequencing Kit, Applied Biosystems, Foster City, CA, U.S.A.) [46]. In addition, the mtDNA D-loop was sequenced directly. The sequence reads were aligned to the revised Cambridge Reference Sequence (rCRS; NC_012920) using Sequencher® version 5.0 sequence analysis software (Gene Codes Corporation, Ann Arbor, MI, U.S.A.). The mtDNA sequences were assigned to haplogroups based on PhyloTree v.17 with HaploGrep2 software [48, 49]. Sequencing was repeated in cases, where haplogroup-defining mutations were missing or where private mutations were found. Phire® Hot Start II DNA polymerase (Thermo Fisher Scientific, Waltham, MA, U.S.A.) was used for all amplifications.

### Variation of interest and mutational load estimates

HaploGrep2 software was used to construct a phylogenetic tree that was based on complete mtDNA sequences and used superhaplogroup L3 as the outgroup [48]. Mutational hotspots m.523_524delAC, m.16182A > C, m.16183A > C, m.16519 T > C and C-insertions at positions m.309, m.315 and m.16193 were not included in the tree. Functional variants were defined as single nucleotide variants in tRNA and rRNA genes, and as variants in protein-coding genes causing amino acid substitutions. The number of such variants was counted in each sequence and the count of rare functional variants included those with minor allele frequency (MAF) less than 1% in MITOMAP (http://www.mitomap.org) and common functional variants included those with MAF ≥ 1%. The variants m.9966G > A and m.2702G > A in subclade N1, m.6261G > A in subclade T2c and m.10398A > G in haplogroup J were removed from all subsequent analysis because of back mutations in these positions. Allele frequencies were based on 30,589 GenBank sequences available at the time of analysis.

APOGEE meta-predictor was used to evaluate the impact of nonsynonymous substitutions [50]. Nonsynonymous variants were deemed nonneutral, if the APOGEE bootstrap mean probability of pathogenicity was greater than 0.5. Mutational load, i.e. the sum of these probabilities in each sequence, was calculated. Probabilities were not estimated for the five nonsynonymous mutations (m.10398G > A, m.8701G > A, m.14766 T > C, m.15326G > A and m.8860G > A) that connect L3 and rCRS in the phylogeny.

### Statistical analysis

Chi-square test (X^2^) was used to evaluate differences in rare and common functional variants between endurance athletes, sprint athletes and the controls, and between haplogroups J and K and the remaining haplogroups. Kruskal–Wallis or Mann–Whitney test was used to assess differences between the groups in continuous variables. IBM® SPSS® Statistics Version 22 software was used.

## Supplementary information

**Additional file 1:** **Figure S1**. Phylogenetic tree of mitochondrial genomes of 141 Finnish athletes and 77 controls (S=sprint athlete; E=endurance athlete; C=control) based on PhyloTree v.17 (48) constructed with HaploGrep2 software (49) and edited using the PDF-XChange Editor V7. Unless an exact base substitution is specified variants are transitions. Insertions are indicated by a decimal point position on the base that the insert follows. Deletions are denoted by “d” after the nucleotide flanking the deletion site. Heteroplasmic positions are indicated by R, S or Y. Variants preceded by @ are assumed back mutations or missing mutations. Colors indicate the following: blue, nonsynonymous mutation; brown, tRNA mutation; green, rRNA mutation.

**Additional file 2:** **Table S1**. A list of rare nonsynonymous functional variants in Finnish athletes and controls.

**Additional file 3:** **Table S2**. A list of rare tRNA functional variants in Finnish athletes and controls.

**Additional file 4:** **Table S3**. A list of rare rRNA functional variants in Finnish athletes and controls.

**Additional file 5:** **Table S4**. Mean mutational load of common and rare nonsynonymous functional variants per subject in Finnish athletes and controls.

**Additional file 6:** **Table S5**. Mean mutational load of common and rare nonsynonymous variants per subject in subjects with haplogroup J and K as compared to other haplogroups.

## Data Availability

The data that supports the findings of this study is available within this paper and its Additional files 1, 2, 3, 4, 5 and 6. Complete mtDNA sequences of athletes have been deposited in NCBI GenBank (http://www.ncbi.nlm.nih.gov/Genbank) under accession numbers MN516554 to MN516694. The sequences from the Finnish controls [46] are available in GenBank under accession numbers AY339402 (C1) to AY339414 (C13), AY339416 (C15) to AY339432 (C31), AY339439 (C38), AY339442 (C41), AY339449 (C48), AY339452 (C51), AY339479 (C78), AY339486 (C85), AY339494 (C93), AY339496 (C95), AY339502 (C101), AY339511 (C110), AY339518 (C117), AY339521 (C120), AY339523 (C122) to AY339532 (C131), AY339534 (C133) to AY339544 (C143), AY339549 (C148), AY339552 (C151), AY339556 (C155), AY339558 (C157), AY339563 (C162), AY339566 (C165), AY339568 (C167), AY339573 (C172), AY339575 (C174), AY339576 (C175), AY339579 (C178), AY339586 (C185), AY339592 (C191) and AY339593 (C192).

## Abbreviations

ATP: Adenosine triphosphate
MELAS: Mitochondrial encephalopathy, lactic acidosis, stroke-like episodes syndrome
mtDNA: Mitochondrial DNA
OXPHOS: Oxidative phosphorylation
rCRS: Revised Cambridge Reference Sequence

## References

[1] Eynon N, Morán M, Birk R, Lucia A (2011). The champions’ mitochondria: is it genetically determined? A review on mitochondrial DNA and elite athletic performance. Physiol Genomics.

[2] Craven L, Alston C, Taylor R, Turnbull D (2017). Recent advances in mitochondrial disease. Annu Rev Genomics Hum Genet.

[3] Anderson S, Bankier AT, Barrell BG, de Bruijn MH, Coulson AR, Drouin J (1981). Sequence and organization of the human mitochondrial genome. Nature.

[4] Wallace DC (2015). Mitochondrial DNA variation in human radiation and disease. Cell.

[5] Niemi AK, Majamaa K (2005). Mitochondrial DNA and ACTN3 genotypes in Finnish elite endurance and sprint athletes. Eur J Hum Genet.

[6] Jastroch M, Divakaruni AS, Mookerjee S, Treberg JR, Brand MD (2010). Mitochondrial proton and electron leaks. Essays Biochem.

[7] Maruszak A, Adamczyk JG, Siewierski M, Sozanski H, Gajewski A, Zekanowski C (2014). Mitochondrial DNA variation is associated with elite athletic status in the polish population. Scand J Med Sci Sports.

[8] Arjmand S, Khaledi N, Fayazmilani R, Lotfi AS, Tavana H (2017). Association of mitochondrial DNA haplogroups with elite athletic status in Iranian population. Meta Gene.

[9] Marcuello A, Martinez-Redondo D, Dahmani Y, Casajus JA, Ruiz-Pesini E, Montoya J (2009). Human mitochondrial variants influence on oxygen consumption. Mitochondrion.

[10] Ballard JW, Pichaud N (2014). Mitochondrial DNA: more than an evolutionary bystander. Funct Ecol.

[11] Gorman GS, Chinnery PF, DiMauro S, Hirano M, Koga Y, McFarland R (2016). Mitochondrial diseases. Nat Rev Dis Primers.

[12] Lakatos A, Derbeneva O, Younes D, Keator D, Bakken T, Lvova M (2010). Alzheimer’s disease neuroimaging initiative. Association between mitochondrial DNA variations and Alzheimer’s disease in the ADNI cohort. Neurobiol Aging.

[13] De Benedictis G, Rose G, Carrieri G, De Luca M, Falcone E, Passarino G (1999). Mitochondrial DNA inherited variants are associated with successful aging and longevity in humans. FASEB J.

[14] Mikami E, Fuku N, Kong QP, Takashi H, Ohiwa N, Murakami H (2013). Comprehensive analysis of common and rare mitochondrial DNA variants in elite Japanese athletes: a case-control study. J Hum Genet.

[15] Liu C, Yang Q, Hwang SJ, Sun F, Johnson AD, Shirihai OS (2012). Association of genetic variation in the mitochondrial genome with blood pressure and metabolic traits. Hypertension.

[16] Fetterman JL, Liu C, Mitchell GF, Vasan RS, Benjamin EJ, Vita JA (2018). Relations of mitochondrial genetic variants to measures of vascular function. Mitochondrion.

[17] Kraja AT, Liu C, Fetterman JL, Graff M, Have CT, Gu C (2019). Associations of mitochondrial and nuclear mitochondrial variants and genes with seven metabolic traits. Am J Hum Genet.

[18] Nachman MW (1998). Deleterious mutations in animal mitochondrial DNA. Genetica.

[19] Rand DM, Kann LM (1998). Mutation and selection at silent and replacement sites in the evolution of animal mitochondrial DNA. Genetica.

[20] James JE, Piganeau G, Eyre-Walker A (2016). The rate of adaptive evolution in animal mitochondria. Mol Ecol.

[21] Zhang F, Broughton RE (2015). Heterogeneous natural selection on oxidative phosphorylation genes among fishes with extreme high and low aerobic performance. BMC Evol Biol.

[22] McManus MJ, Picard M, Chen HW, De Haas HJ, Potluri P, Leipzig J (2019). Mitochondrial DNA variation dictates expressivity and progression of nuclear DNA mutations causing cardiomyopathy. Cell Metab.

[23] O'Keefe H, Queen RA, Meldau S, Lord P, Elson JL (2018). Haplogroup context is less important in the penetrance of mitochondrial DNA complex I mutations compared to mt-tRNA mutations. J Mol Evol.

[24] Ye K, Lu J, Ma F, Keinan A, Gu Z (2014). Extensive pathogenicity of mitochondrial heteroplasmy in healthy human individuals. Proc Natl Acad Sci U S A.

[25] Payne BA, Wilson IJ, Yu-Wai-Man P, Coxhead J, Deehan D, Horvath R (2013). Universal heteroplasmy of human mitochondrial DNA. Hum Mol Genet.

[26] Hutchin T (1999). Sensorineural hearing loss and the 1555G mitochondrial DNA mutation. Acta Otolaryngol.

[27] Taylor RW, Turnbull DM (2005). Mitochondrial DNA mutations in human disease. Nat Rev Genet.

[28] Grady JP, Pickett SJ, Ng YS, Alston CL, Blakely EL, Hardy SA (2018). mtDNA heteroplasmy level and copy number indicate disease burden in m.3243A>G mitochondrial disease. EMBO Mol Med.

[29] Dvorakova V, Kolarova H, Magner M, Tesarova M, Hansikova H, Zeman J (2016). The phenotypic spectrum of fifty Czech m.3243A>G carriers. Mol Genet Metab.

[30] Wang L, Wang X, Cai X, Qiang R (2019). Study of mitochondrial DNA A1555G and C1494T mutations in a large cohort of women individuals. Mitochondrial DNA A DNA Mapp Seq Anal.

[31] Elliott HR, Samuels DC, Eden JA, Relton CL, Chinnery PF (2008). Pathogenic mitochondrial DNA mutations are common in the general population. Am J Hum Genet.

[32] Lehtonen MS, Uimonen S, Hassinen IE, Majamaa K (2000). Frequency of mitochondrial DNA point mutations among patients with familial sensorineural hearing impairment. Eur J Hum Genet.

[33] Majamaa K, Moilanen JS, Uimonen S, Remes AM, Salmela PI, Kärppä M (1998). Epidemiology of A3243G, the mutation for mitochondrial encephalomyopathy, lactic acidosis, and strokelike episodes: prevalence of the mutation in an adult population. Am J Hum Genet.

[34] Thompson MA (2017). Physiological and Biomechanical Mechanisms of Distance Specific Human Running Performance. Integr Comp Biol.

[35] Niemi AK, Hervonen A, Hurme M, Karhunen PJ, Jylha M, Majamaa K (2003). Mitochondrial DNA polymorphisms associated with longevity in a Finnish population. Hum Genet.

[36] Kenney MC, Chwa M, Atilano SR, Pavlis JM, Falatoonzadeh P, Ramirez C (2013). Mitochondrial DNA variants mediate energy production and expression levels for CFH, C3 and EFEMP1 genes: implications for age-related macular degeneration. PLoS One.

[37] Sazanov LA (2015). A giant molecular proton pump: structure and mechanism of respiratory complex I. Nat Rev Mol Cell Biol.

[38] Ruiz-Pesini E, Wallace DC (2006). Evidence for adaptive selection acting on the tRNA and rRNA genes of human mitochondrial DNA. Hum Mutat.

[39] Ji F, Sharpley MS, Derbeneva O, Alves LS, Qian P, Wang Y (2012). Mitochondrial DNA variant associated with Leber hereditary optic neuropathy and high-altitude Tibetans. Proc Natl Acad Sci U S A.

[40] Kang L, Zheng HX, Zhang M, Yan S, Li L, Liu L (2016). MtDNA analysis reveals enriched pathogenic mutations in Tibetan highlanders. Sci Rep.

[41] Jain IH, Zazzeron L, Goli R, Alexa K, Schatzman-Bone S, Dhillon H (2016). Hypoxia as a therapy for mitochondrial disease. Science.

[42] Suissa S, Wang Z, Poole J, Wittkopp S, Feder J, Shutt TE (2009). Ancient mtDNA genetic variants modulate mtDNA transcription and replication. PLoS Genet.

[43] Torroni A, Petrozzi M, D'Urbano L, Sellitto D, Zeviani M, Carrara F (1997). Haplotype and phylogenetic analyses suggest that one European-specific mtDNA background plays a role in the expression of Leber hereditary optic neuropathy by increasing the penetrance of the primary mutations 11778 and 14484. Am J Hum Genet.

[44] Rose G, Passarino G, Carrieri G, Altomare K, Greco V, Bertolini S, Bonafè M, Franceschi C, De Benedictis G (2001). Paradoxes in longevity: sequence analysis of mtDNA haplogroup J in centenarians. Eur J Hum Genet.

[45] Raule N, Sevini F, Li S, Barbieri A, Tallaro F, Lomartire L (2014). The co-occurrence of mtDNA mutations on different oxidative phosphorylation subunits, not detected by haplogroup analysis, affects human longevity and is population specific. Aging Cell.

[46] Finnilä S, Lehtonen MS, Majamaa K (2001). Phylogenetic network for European mtDNA. Am J Hum Genet.

[47] Meinilä M, Finnilä S, Majamaa K (2001). Evidence for mtDNA admixture between the Finns and the Saami. Hum Hered.

[48] van Oven M, Kayser M (2009). Updated comprehensive phylogenetic tree of global human mitochondrial DNA variation. Hum Mutat.

[49] Kloss-Brandstatter A, Pacher D, Schönherr S, Weissensteiner H, Binna R, Specht G (2011). HaploGrep: a fast and reliable algorithm for automatic classification of mitochondrial DNA haplogroups. Hum Mutat.

[50] Castellana S, Fusilli C, Mazzoccoli G, Biagini T, Capocefalo D, Carella M (2017). High-confidence assessment of functional impact of human mitochondrial non-synonymous genome variations by APOGEE. PLoS Comput Biol.

