# Supplementary material for: Analysis of functional variants in mitochondrial DNA of Finnish athletes
Source: BMC Genomics. 2019 Oct 29;20:784. doi: 10.1186/s12864-019-6171-6 (PMC6819560; doi:10.1186/s12864-019-6171-6)
Supplement: Supplementary file 1 — Additional file 1: Figure S1. Phylogenetic tree of mitochondrial genomes of 141 Finnish athletes and 77 controls (S=sprint athlete; E=endurance athlete; C=control) based on PhyloTree v.17 (48) constructed with HaploGrep2 software (49) and edited using the PDF-XChange Editor V7. Unless an exact base substitution is specified variants are transitions. Insertions are indicated by a decimal point position on the base that the insert follows. Deletions are denoted by “d” after the nucleotide flanking the deletion site. Heteroplasmic positions are indicated by R, S or Y. Variants preceded by @ are assumed back mutations or missing mutations. Colors indicate the following: blue, nonsynonymous mutation; brown, tRNA mutation; green, rRNA mutation. [file 12864_2019_6171_MOESM1_ESM.pdf]

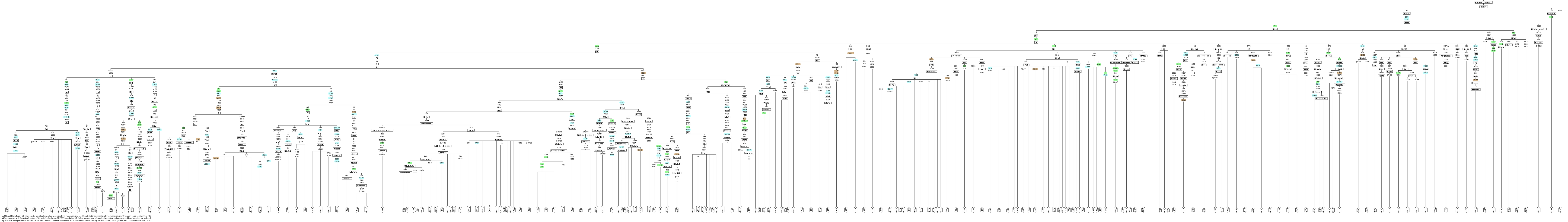

Additional file 1. Figure S1. Phylogenetic tree of mitochondrial genomes of 141 Finnish athletes and 77 controls (S=sprint athlete; E=endurance athlete; C=control) based on PhyloTree v.17 (48) constructed with HaploGrep2 software (49) and edited using the PDF-XChange Editor V7. Unless an exact base substitution is specified variants are transitions. Insertions are indicated by a decimal point position on the base that the insert follows. Deletions are denoted by "d" after the nucleotide flanking the deletion site. Heteroplasmic positions are indicated by R, S or Y. Variants preceded by @ are assumed back mutations or missing mutations. Colors indicate the following: blue, nonsynonymous mutation; brown, tRNA mutation; green, rRNA mutation.
